# Integrating Human-Centered Design to Advance Global Health: Lessons From 3 Programs

**DOI:** 10.9745/GHSP-D-21-00279

**Published:** 2021-11-29

**Authors:** Emily Blynn, Emily Harris, Melanie Wendland, Courtney Chang, Dyness Kasungami, Monisha Ashok, Metsehate Ayenekulu

**Affiliations:** a Independent consultant, Washington, DC, USA.; b U.S. Agency for International Development, Washington, DC, USA.; c Sonder Collective, Helsinki, Finland.; d Ideo.org, San Francisco, CA, USA.; e John Snow Research & Training Institute, Inc., Boston, MA, USA.; f Population Services International, Ethiopia, Addis Ababa, Ethiopia.

## Abstract

**Background:**

Human-centered design (HCD) is increasingly being used as a complementary approach to traditional global health methods due to its ability to bring new ideas to entrenched problems, integrate multiple stakeholder perspectives, and bring in a strong human lens among other advantages. To reap these benefits, public health and design practitioners in global health programs can learn from the early experiences of integrating HCD to advance these efforts.

**Objective:**

This article distills lessons gathered from 3 programs leveraging HCD to advance global health programming: (1) the “V” program which used an HCD approach to reframe the once-a-day HIV prevention pill from a potentially stigmatizing medicine into empowering self-care; (2) the Adolescents 360 program which integrated HCD to create a service for adolescent girls to access contraception in Ethiopia and to scale this offering nationwide; and (3) Reimagining TA which used HCD to help shift perceptions around traditional technical assistance models to one of co-creation, defining a new approach for non-financial support for health systems strengthening.

**Findings:**

To inform global health programs that are considering employing an HCD approach, lessons learned are distilled into 3 categories: (1) planning: considerations for problem definition and project scoping to allow for flexibility and selection of appropriate methods; (2) engaging: reflections on the means to productively engage different stakeholder groups to build alignment, understanding, and buy-in; (3) applying: adoption of new ways of working during implementation to best take advantage of the benefits of HCD while promoting long-term program sustainability and learning.

**Conclusion:**

These lessons represent an important step on the pathway to demonstrate the contributions of HCD to improving the effectiveness of health programs at a time when the global health community needs the most robust set of tools possible to meet the demands of our current pandemic context and beyond.

## Abstract (precis)

Lessons from 3 global health programs indicate that human-centered design (HCD) holds great potential for developing more tailored, impactful, and sustainable products and services to improve health and well-being. However, to take advantage of the full benefits of HCD, global health practitioners need to intentionally design and implement programs differently from typical health programs that do not incorporate design.

## Teaser

**Key Messages:**

Reflections from 3 global health programs using human-centered design (HCD) offer 3 categories of lessons for those considering similar approaches:

- Planning while considering the needs of both traditional global health and HCD approaches
- Engaging key stakeholders to build understanding, alignment, and buy-in from the outset
- Applying approaches differently from the way both designers and global health actors are accustomed to working to promote long-term program sustainability and learning

**Key Implications:**

- If implemented appropriately, integrating HCD into global health programming can produce a virtuous cycle between co-creation, stakeholder buy-in, and quality of outputs. The more that programs engage stakeholders in an inclusive, participatory process, the greater their continued willingness and motivation. This in turn allows for more iteration and higher quality, better-tailored outputs that are more likely to be sustainably used and scaled.
- To engender this virtuous cycle, programs that incorporate an HCD approach will need to be scoped differently than traditional global health programs (e.g., more flexible timelines; dedicated budget for implementation and capacity building, etc.).
- Because stakeholders may perceive a higher risk of failure with a new approach, proponents of HCD are faced with a substantial burden of evidence to persuade actors to consider its benefits. However, traditional global health actors should consider alternative approaches to measuring HCD’s contributions, including perceived end user value.

## BACKGROUND

Despite the billions of dollars expended for global health in the last decade, the global community remains far from achieving many of the ambitious Global Goals for Sustainable Development (SDGs) for health.^1^ Global malaria cases and deaths have plateaued, progress toward ending preventable maternal deaths has slowed, and despite remarkable global progress on under-5 mortality, stark disparities persist between regions.^2^^,^^3^ Over the past year, advancement toward these goals has been further eroded due to the primary and secondary effects of COVID-19. For example, due to interruptions in service delivery, immunization coverage has fallen to rates last experienced in the 1990s.^4^ Global health programming seeks to address these challenges.^5^^–^^7^ In particular, the learnings in this article are targeted toward large, multistakeholder partnerships—often constituting a combination of a funder, an implementing partner(s), and local government—implementing public health interventions in low- and middle-income countries at a national or international scale and reaching a substantial proportion of the target population.^8^^–^^10^ Human-centered design (HCD) is increasingly being used in global health programs as a complement to traditional public health methods due to its ability to bring new ideas to entrenched problems, inclusively integrate multiple stakeholder perspectives, and bring in a strong human lens, among other advantages.^11^^–^^21^

Recognizing that while HCD has its limitations, this article seeks to illuminate reflections and lessons learned from 3 global health programs that sought to integrate this approach. Reflecting on these experiences, this article can benefit both traditional global health funders and implementers seeking to introduce HCD into their work and/or improve the effectiveness of existing projects, as well as designers seeking to use their HCD skills to advance global health. These reflections aim to help both groups successfully integrate their different approaches to reap the benefits of HCD in the context of global health programs.

The reflections in this article aim to help both global health practitioners and designers successfully integrate their different approaches to reap the benefits of HCD in the context of global health programs.

## INTERVENTIONS

The 3 programs discussed in this article all integrated HCD into traditional global health programming, but they vary in important ways that can illuminate learnings for a broad range of global health programs (Table).

**TABLE.** Featured Program Characteristics and Approaches to HCD Integration

	**Project V**	**A360**	**Reimagining TA**
Scope	Product	Program	System
Goal	Reframe oral PrEP to increase demand and adherence	Increase demand and access to modern contraception	Inspire and plant a seed for system change for improved TA
Target audience	Adolescent girls and influencers (parents, partners, health care workers)	Married adolescent girls	Global and in-country users, buyers, and implementers of TA
HCD dose (i.e., Design as an ingredient;Design as a spark; Design end-to- end)^22^	Design end-to-end	Design as an ingredient	Design as a spark
Duration	South Africa: 2015–2018Zimbabwe: 2019–present	2016–2020	2018–2020
Application of HCD to advance global health programming	How HCD can reframe a product (shifting the perception of oral PrEP from medicine to self-care) to advance an HIV prevention service delivery program	How HCD can be used to define a new service delivery solution (“Smart Start”) that can be adopted by the ministry of health and rolled out countrywide	How HCD can be used to redefine program planning and delivery itself (technical assistance) in the context of global health programs
HCD methods employed	Market (literature review, stakeholder interviews) and design research (immersions, workshops), synthesis (user journey mapping), prototyping	Design research, synthesis, prototyping	Design research (stakeholder interviews), co-creation workshops, and design sprints
Outputs	Four-pillar implementation approach drawing inspiration from fashion and beauty brands, including a starter kit styled after a makeup bag that includes a lip balm shaped pill case that silences rattling	Smart Start, a program that uses goal setting and financial planning as an entry point to discussing the role of contraception with young married couples.	1. Nine critical shifts that need to occur to transform the current TA system. These shifts are a bridge between the identified challenges of current approaches and the future vision 2. Twenty design principles of good TA for global and local stakeholders
Funders and partners	South Africa: PEPFAR-supported, USAID-managed via Project EMOTION Zimbabwe: PEPFAR-supported, USAID-managed via Engage Design, in partnership with PSI, PZAT^a^	PSI, IDEO.org, Center on the Developing Adolescent at UC Berkeley, Bill & Melinda Gates Foundation, Children’s Investment Fund Foundation	Bill & Melinda Gates Foundation, Child Health Task Force Secretariat (through JSI), Sonder Collective

Abbreviations: HCD, human-centered design; JSI, John Snow Inc.; PZAT, Pangaea Zimbabwe AIDS Trust; PEPFAR, U.S. President’s Emergency Plan for AIDS Relief; PrEP, pre-exposure prophylaxis; PSI, Population Services International; TA, technical assistance, UC; University of California; USAID, U.S. Agency for International Development.

a V Zimbabwe was created in collaboration with adolescent girls and young women, health care workers, and a global network of partners led by Engage Design—a human-centered design partnership including McKinsey & Company, Matchboxology, and PATH—alongside PSI Zimbabwe, Pangaea Zimbabwe AIDS Trust, and the Zimbabwe Ministry of Health and Child Care. V Zimbabwe is infused with the spirit of EMOTION, a CONRAD-led partnership with IDEO, Matchboxology, Abt Associates, CAPRISA, and Instant Grass, that launched V (https://www.conrad.org/launchingv/). V Zimbabwe was made possible by the support of the American people through the United States Agency for International Development (USAID) under the U.S. President’s Emergency Plan for AIDS Relief (PEPFAR) through EngageDesign contract 7200AA18M00011 and EMOTION Cooperative Agreement AID-OAA-A-15-00033.

### V

In 2015, the U.S. Agency for International Development (USAID) engaged a consortium of partners led by CONRAD, and including IDEO and Matchboxology, to identify a new approach to offering oral pre-exposure prophylaxis (PrEP) to adolescent girls and young women (AGYW). While access to oral PrEP is scaling up across sub-Saharan Africa, low rates of initiation and retention continue to limit the progress toward ambitious, global goals for AGYW.^23^ USAID hypothesized that an HCD approach could help program implementers better understand what AGYW want when it comes to HIV prevention, shifting from the more traditional “demand creation” to “desire creation.”

Formative research in South Africa included user journey mapping and highlighted a negative, overmedicalized experience of PrEP. Based on this research, “V” was co-created with AGYW through prototyping and testing, resulting in a radically reframed PrEP experience from a potentially stigmatizing medicine into empowering self-care. V is introduced through brand ambassadors and initiated using a starter kit that mimics a makeup bag, including a small pill case that silences pill rattling and can be shown off or disguised as a lip balm case.

In 2019, USAID engaged the Engage Design consortium—led by McKinsey Design, Matchboxology, and research partner PATH—to partner with USAID/Zimbabwe’s leading HIV prevention service delivery partners to adapt V for program implementation. Due to COVID-19, implementation was paused but has recently resumed, with results expected in late 2021.

### Adolescents 360 Ethiopia

Adolescents 360 (A360) is a 4-year initiative (2016–2020) to increase adolescent girls’ access to and demand for modern contraception in Nigeria, Ethiopia, and Tanzania. A360 is implemented by Population Services International (PSI) and in partnership with IDEO.org, the Center on the Developing Adolescent at the University of California, Berkeley, and the Society for Family Health Nigeria, and cofunded by the Bill & Melinda Gates Foundation and the Children’s Investment Fund Foundation. A360 used HCD alongside other disciplines of social marketing, developmental neuroscience, and anthropology to develop innovative country-specific interventions through an iterative process of researching, testing, prototyping, and piloting ideas with girls and other stakeholders. The first 2 years were dedicated to program design and optimization, while the second 2 years were focused on implementation and scale-up.

The HCD approach applied led to the development of Smart Start, an intervention that uses financial planning as an entry point to engage young married couples in planning their futures and reaching financial stability, positioning contraception as a tool to achieve their self-defined goals. Smart Start supports girls aged 15–19 years and their partners to understand the resources they will need for the families they desire.

As of September 2020, Smart Start has reached 76,480 girls in Ethiopia, with 35,887 (79% of eligible girls who are not pregnant and not using contraception) of those voluntarily adopting a form of modern contraception. It is currently being implemented in 1,070 sites across the country and the Ministry of Health (MOH) is embedding it in the national health extension worker program. PSI has also introduced and adapted Smart Start for adolescent girls in Mozambique.

### Reimagining Technical Assistance

Reimagining Technical Assistance (Reimagining TA) was a 2-year project (2018–2020) funded by the Bill & Melinda Gates Foundation and implemented by the Child Health Task Force Secretariat through John Snow Research & Training Institute (JSI R&T) and Sonder Collective. TA has been criticized for failing to align with country priorities or coordinate with governments and other stakeholders, often focusing on short-term wins, and lacking systematic approaches to solve public health challenges.^24^^–^^26^ The project goals were to invite in-country TA stakeholders to engage in a co-creation process to map current barriers and opportunities in how TA is designed and delivered; co-create a shared vision and concepts for the future of TA; and test, iterate, and develop new models for TA in Nigeria and the Democratic Republic of Congo (DRC). To achieve these goals, the project team adopted an integrated approach that combined HCD, co-creation, and systems thinking. The HCD aspects of the process involved activities to understand the human experience of TA, including attitudes, motivations, and behaviors, as well as social determinants, including social norms and group identity. In each country, the project used HCD and co-creation processes that included workshops, design sprints, and stakeholder interviews. The system's thinking included mapping the journey of TA planning and delivery that recognized interactions among international, several national government departments, and subnational actors as opposed to a single project addressing a technical intervention.

The project co-creation teams identified 9 critical shifts and 20 design principles of good TA for global and local stakeholders, which serve as a platform for alignment, inspiration, and action toward a re-imagined TA.

## LESSONS LEARNED ACROSS PROGRAMS

Reflections on these projects hold lessons for global health practitioners and designers seeking to advance global health programming through HCD. Learnings can be broadly distilled into 3 categories: (1) planning: considerations for problem definition and project scoping to satisfy integration of HCD methods; (2) engaging: reflections on the means to productively engage different stakeholder groups to build alignment, trust, and buy-in; and (3) applying: adoption of new ways of working during implementation to best exploit the benefits of HCD while promoting long-term program sustainability and learning (Figure).

**FIGURE:**
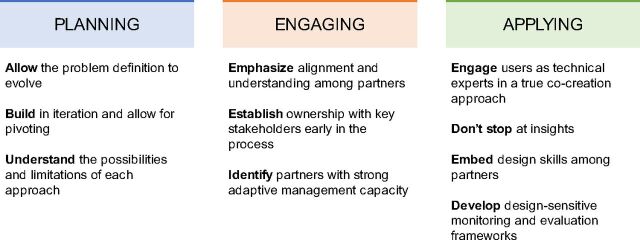
Summary of Lessons Learned Across 3 Projects on Integrating Human-Centered Design Into Global Health Programming

### 1. Planning

Across the 3 programs, early project planning phases were critical to realize the benefits of HCD methods. Traditional global health practitioners needed to adapt their approaches to consider the flexibility, iterative nature, and sometimes elongated time frames that HCD requires to produce robust results. Similarly, designers needed to invest time during the planning phase to understand how design could best add value.

Traditional global health practitioners needed to adapt their approaches to consider the flexibility, iterative nature, and sometimes elongated time frames that HCD requires to produce robust results.

#### Allow the Problem Definition to Evolve

Because HCD is a creative problem-solving approach, projects must have a clear, baseline problem definition that is developed and refined through research with end users and other stakeholders. However, experiences of these programs also suggest that continuing to iterate on the problem definition throughout the project can advance better outputs.

For example, in A360 Ethiopia, the program initially sought to achieve 121,000 new users of contraception, aged 15–19 years over 4 years. However, after the insights gathering phase, the team uncovered 3 different segments in this age bracket that would require different solutions: unmarried girls in school, young married adolescents, and young adolescent mothers. This fueled a conversation about redefining the scope of the project, the user segment, and the appropriate targets, which ultimately laid the foundation for designing a highly tailored program for one of these segments.

V’s formative research in South Africa suggested that V would be most impactful if it was positioned as a consumer brand. Consequently, the materials were designed with a look and feel that was intentionally distinct from traditional public health communication materials. However, when translating the product for use in Zimbabwe, users wanted V to be linked to current oral PrEP offerings through public health programs to avoid confusion. For future replication, the team recommended that a rapid, low-resource validation within an existing program be conducted first. This would allow the designers leading the HCD process to facilitate the initial phase of problem definition based on an understanding of the specific country and program context.

In Reimagining TA, the timeline, project approach, and funding had been allocated to the implementing partners with the assumption of a single, broad problem definition. However, though all actors agreed that the TA delivery was broken and needed to be reconceived, during the initial in-country workshops the design team discovered that "the problem" with TA was more complex and nuanced than previously understood. For example, while there was consensus that the current timeframes for TA projects were not appropriate, funders preferred shorter-term TA projects and near-term results, while governments wanted several years of TA to strengthen the underlying capacity of the health system to deliver long-term and sustainable results. These multiple and partly diverging problem definitions between global, national, and local stakeholders were at odds with the ability to undertake a fast HCD process. A more collaborative problem definition phase could have helped establish a clear, shared intent for the work and identify solutions more suited to the needs of stakeholders at all levels.

#### Build in Iteration and Allow for Pivoting

The HCD process is rooted in deep inquiry and open exploration to surface new insights and develop innovative products or processes together with the users of those solutions. As a result, the prototype or proposed solution will rarely be the one to scale. This process of iteration and adaptation can often take more time and can be in tension with the more linear structure of traditional global health programs. Recognizing this tension and building in more time and resources for a rigorous, iterative HCD process can enable global health programs to reap the benefits of HCD.

Building in more time and resources for a rigorous, iterative HCD process can enable global health programs to reap the benefits of HCD.

From the beginning, A360 Ethiopia had a generous timeline and expectation for program adaptation, which allowed the initiative to be successful at scale. In the solution design phase, there were 3 rounds of rapid prototyping before a 6-month pilot. Through these iterations, the team quickly validated the value proposition of financial planning for young couples, experimented with how to best communicate the relationship between financial and family planning, and ultimately, refined the delivery model for this intervention for married adolescents in rural Ethiopia. In the second year, even after the Smart Start financial planning curriculum was launched, a second short design sprint helped condense the curriculum from 60 pages and 1 hour to 12 pages and 15 minutes, making it much easier for health extension workers to integrate these messages into their work.

Similarly, in Reimagining TA, the team found that conducting a design process within a complex system requires time. While the donor originally allocated funding for an 8-month timeline, the project ultimately took 24 months for several reasons. First, the project encountered logistical constraints. Co-creation workshops required direct engagement with country-level stakeholders, particularly government officials, who had limited availability, and that was exacerbated by personnel turnover in key roles post-elections. Second, in the case of the DRC, the first round of prototyping surfaced problems at the community level that resulted from poorly designed TA, including lack of consideration of the burden of out-of-pocket health costs on the families, lack of dialogue with community leaders, and duplication of activities in the field. The team decided that a second round of prototyping was needed to identify the root, systems-level problems. Third, once the timeline began to get delayed, amendments to the pre-defined project plan and budget took additional time.

To address these challenges, the project concept should be codesigned by the funder, the content and process experts, and the collaborating government entities. With all key stakeholders at the table, expectations would be clarified, roles and responsibilities agreed upon, accountability mechanisms established, and funding allocated based on a realistic timeline and level of effort. Ultimately, a timeline that allows for co-creation of the vision, iteration, and exploration throughout the process is crucial to consider for successfully integrating HCD into health programs.

A timeline that allows for co-creation of the vision, iteration, and exploration throughout the process is crucial to consider for successfully integrating HCD into health programs.

For A360 Ethiopia, navigating the institutional review board (IRB) process surfaced tensions between HCD’s discovery-driven and public health’s pre-articulated research approach. For public health practitioners, IRB approval is required when deemed necessary to protect the human subjects of research. HCD practitioners are accustomed to open and fluid lines of inquiry with users to discover new and unexpected insights and often utilize market research approaches that are exempt from IRB requirements. A360 Ethiopia pursued IRB approval in line with the principles of its Commitment to Ethics in Youth-Powered Program Design. Initial IRB feedback stated that A360 did not have an adequate sample size and had to change the wording of questions and limit their methods to a specific set of 9 approaches. The requirement to submit the design research plan 6 to 9 months in advance of project launch also limited the design team’s ability to adapt their methods as needed throughout the process. The IRB process did help pre-articulate the lines of inquiry that would truly advance programming while eliminating those that are potentially extractive and not relevant to programming. A360’s experience demonstrates how adhering to ethical standards in design can be achieved while maintaining the uniquely iterative nature of design research. For example, future HCD practitioners can learn from A360’s approach of including in the final IRB application a range of approaches, such as “inquiry questions may include” “we will speak to a maximum of X people.” By pursuing discovery-driven approaches that also promote documenting methods and results, HCD practitioners can advance the diffusion of potentially catalytic approaches such as design, while ensuring common ethical standards across the global health and design communities.^27^

#### Understand the Possibilities and Limitations of Each Approach

Part of the value of embedding HCD in global health programming is the opportunity to use it in combination with traditional public health methods and other complementary methodologies. Each of the program experiences highlighted the importance of identifying the specific areas where HCD can add value and where other approaches can be useful (and where these have limitations). Further detail on the value of design as a complementary methodology is detailed in a separate article in this supplement.^28^

The experience of V demonstrates how HCD can be used to build on existing knowledge and derive new solutions. V’s formative findings reinforced well-documented opportunities (e.g., how female-controlled HIV prevention products can capitalize on the concept of control for young women struggling with a lack of agency) and challenges (e.g., how discretion is needed to combat the stigma associated with a product whose users are perceived as engaging in risky sexual behavior).^29^ V’s solutions build on well-established social marketing principles, such as linking health products with desirable private sector positioning and messaging, and well-researched interventions, such as pill cases’ ability to improve adherence.^30^ HCD’s unique contribution was the ability to build on these known truths and design a new solution tailored to the needs of the target segment. If successful, V will demonstrate how aligning oral PrEP with a beauty product promotes both desirability and behavior change (e.g., by taking advantage of existing wellness routines to promote adherence), discretion and control (e.g., through its ability to be concealed as a nonmedical consumer product), practicality and delight (e.g., by offering a pill case with design elements that meet young women’s unique needs in a novel form not available at their local pharmacy).

In Reimagining TA, the team found that with a project as complex as reimagining a system of service delivery at global scale, other approaches such as systems thinking and behavioral science would have been useful in addition to HCD. The project’s HCD approach centered around mapping the human experience of TA, the user needs of different actors involved, and journeys through different TA programs. This helped to understand people, their interactions, and the complexity of those interactions. However, a systems-thinking approach could have helped better identify linkages and power dynamics in these systems that could be targeted for change. In addition, reimagining a system involves changing the behavior of the people in that system. The HCD approach in this project did not apply a behavioral science approach to analyze behavior to propose a behavior change strategy.

### 2. Engaging

While implementing these projects, both traditional global health actors and designers found that they needed to adapt their traditional ways of working to successfully collaborate on integrating HCD into global health programming. Teams found an initial start-up phase useful to build alignment, understanding, and trust among the different groups involved. They also found that establishing ownership with MOHs, implementing partners, and other key stakeholders early in the process was particularly important for sustainability, as was identifying partners with strong existing capacity in adaptive management.

#### Emphasize Alignment and Understanding Among All Partners

Embedding HCD in a global health program requires a shift in the way both global health practitioners and designers are accustomed to working. The 3 projects thus noted that a start-up alignment phase would be helpful to clearly delineate roles and responsibilities of each partner in the HCD process and familiarize traditional global health actors with the baseline components of the HCD approach.

A start-up alignment phase would be helpful to clearly delineate roles and responsibilities of each partner in the HCD process and familiarize traditional global health actors with the baseline components of the HCD approach.

In Reimagining TA, no such start-up phase was built in for Sonder and JSI to align on process and approach. The design team had to orient the JSI team to the HCD methodology and approach while simultaneously kicking off activities in-country, as the JSI team had not worked with HCD before. Equally, the design team needed time to understand the country’s context and effective ways of working with the national governments. The lack of time to align on how to integrate a consultant-led design process into the global health program exposed a lack of mutual understanding in terms of ways of working. This created confusion, mutual questioning of the project approach, and a lack of clarity around roles and responsibilities. Had there been an initial codesign and planning phase for the 2 organizations to deeply engage with the planned approach and methodology, reflect on the country context and its impact on the process, build mutual understanding and trust, map out roles and responsibilities carefully, and align the timeline accordingly, the project would have been delivered in a more targeted, streamlined, and effective manner.

Conversely, V invested time and resources to transition formative research conducted in South Africa to testing, adapting, and integrating the approach into HIV programming in Zimbabwe. The prototypes of the V starter kits and distribution plans developed in South Africa were not automatically transferable to a new context given the procurement realities of supplying large, U.S. government-funded programs. Ultimately, implementing V in Zimbabwe required procurement through an existing U.S. government supply chain mechanism focused on pharmaceutical product procurements, which required a competitive contracting process. The start-up planning phase in Zimbabwe was essential to transition from prototyping to program implementation. In addition, because of the investment of time and effort in procuring through an existing U.S. government supply chain mechanism, V is now much more accessible for other country teams who might want to procure it.

#### Establish Ownership With Key Stakeholders Early in the Process

In traditional global health programming, partnerships are already regarded as important, particularly partnerships with MOHs, to build buy-in and promote sustainability. However, when adding in a less familiar HCD approach, generating early buy-in was even more important.

The key to A360 Ethiopia’s sustainability was its early and flexible partnership with the MOH. Because A360’s objectives closely aligned with the National Adolescent and Youth Health Strategy targets for reducing teen pregnancy, the MOH participated early in A360’s proposal development. While the MOH was skeptical of the longer timeline and programmatic delays that an intensive co-creation approach would entail, inviting young designers to present the insights and opportunities that emerged from design research ultimately helped convince the MOH of the approach. It was a powerful signal, coming directly from their constituents, about what change was needed and whose voices needed to be heard to address teen pregnancy. Design afforded the rare and insightful opportunity for the MOH to hear directly from constituents that they may not have had otherwise.

For Reimagining TA, a lack of strong government buy-in affected the quality and speed of the co-creation process. Given the limited timeline for securing government buy-in, the government accepted the initiative without full awareness of and commitment to the level of engagement needed for a true co-creation process. As the project was one among many donor-funded projects competing for stakeholder time and attention, it lost out against other priorities that were included earlier in the country’s operational plans. As previously noted, including the country governments in co-creating the project concept could have helped to achieve greater buy-in.

#### Identifying Partners With Strong Adaptive Management Capacity

The HCD process is based on the rapid development of prototypes and iteration, which means that the output of a project is never truly “finished.” The experiences of these projects suggest that partners with strong adaptive management skills (i.e., the ability to make adjustments based on new information) are those best suited to continue to test, learn, and improve the solution to bring it to scale.

The experiences of these projects suggest that partners with strong adaptive management skills are best suited to continue to test, learn, and improve the solution to bring it to scale.

For the Reimagining TA project, the Technical Assistance Hub (TA Hub), an initiative of the Bill & Melinda Gates Foundation Nigeria country office and implemented by DAI Nigeria, was able to adopt, adapt, and use the project outputs. The TA Hub is an independent nonprofit organization that coordinates the delivery of comprehensive technical assistance and institutional strengthening support to state governments in Nigeria. DAI staff participated in the Reimagining TA process as co-creation team members and were later able to apply the reimagined TA approach. Now, all partners applying to provide TA to state governments in Nigeria with funding from the TA Hub are required to demonstrate how they will apply the critical shifts and principles of good TA. Identifying a partner with the capacity to adapt and iterate on the project outputs was critical to the project’s implementation.

For A360 Ethiopia, a key factor for success was PSI Ethiopia building an institutional culture of adaptive management, which allowed the Smart Start solution to scale beyond the initial design phase. After the initial work was completed, the PSI Ethiopia team created an internal “Learning and Insights Team,” which continued to practice design and adaptive management, including routine data monitoring, conducting focus groups, and taking action to adjust key program components as needed when scaling to a new region or training a new tier of health workers. This has allowed them to quickly identify and adjust program components when scaling to a new region or training a new tier of health workers.

### 3. Applying

When putting HCD into practice in the context of global health programming, both designers and traditional global health stakeholders found they needed to adapt their typical approaches to implement an integrated program. First, to develop outputs tailored to the needs of the end user, teams found it critical to engage in a true co-creation process with end users and other stakeholders throughout the project, with each participant adding equal value; this represented a shift for more traditional global health actors. Second, though some HCD projects end with insight generation, in this work, teams found it necessary to push toward implementation of the product or service to promote uptake of the final outputs. To ensure the program can continue once the core work has concluded, teams found that building HCD capacity among the consortium of partners allowed them to continue to iterate and adapt the solutions based on the needs of the target population. Finally, when designing monitoring and evaluation (M&E) frameworks for these programs, either traditional global health actors or designers needed to adapt their approach to identify a set of indicators well-suited to the integrated approach.

#### Engage Users as Technical Experts in a True Co-creation Approach

Traditional global health programs and HCD projects vary in the degree to which they include and embed input from end users and other key stakeholders. In traditional global health programming, consulting end users and including other key stakeholders at key decision points is not a new concept. However, experiences from these projects suggest that it is necessary to engage in a true co-creation process, involving the end users and stakeholders at each stage of the work, rather than a more prescriptive, donor-recipient relationship.

Experiences from these projects suggest that it is necessary to engage in a true co-creation process, involving the end users and stakeholders at each stage of the work, rather than a more prescriptive, donor-recipient relationship.

A key component of A360 Ethiopia was co-creation of solutions with young Ethiopian designers. During prototyping young people played a significant role in identifying the most resonant ideas, resolving conflicting feedback in testing, and improving ideas to be more contextually appropriate. For example, when discussing the cost of having a baby, a young designer proposed expressing it in the number of goats, rather than the local currency, which was a more relatable concept for the pastoralist community. A second benefit to co-creation was that having young designers as a core part of solution development gave Smart Start credibility among the MOH stakeholders who saw these young people as their constituents. Engaging youth as team members in this way requires dedicated financial resources, human resources, and time, which may often be at odds with short-term efficiency; yet it was central to the success of the project’s outputs and long-term sustainability.

In the case of the Reimagining TA project, the degree of co-creation varied by project stage and country context. The project launch represented a more prescriptive approach; countries were preselected by the funder, and country stakeholders were only involved after funding was approved. Country governments were not involved in developing the problem definition in the proposal to determine if the related process and approach would be meaningful and needed in their country. As a result, throughout the project, it was difficult to achieve stakeholders’ buy-in and commitment, despite intentionally adopting a more collaborative co-creation approach in later project phases. In Nigeria, inconsistent stakeholder engagement in the workshops resulted in fragmented insights and low commitment to the process and the solutions. Conversely, in the DRC, the co-creation team was consistent and engaged throughout the whole process, which resulted in better adoption and implementation of the solutions. By the end of the project, the DRC team was ready and willing to take independent ownership of the next steps. Involving stakeholders not as participants but as co-creators requires global health funders and other traditional stakeholders to engage in a more inclusive process from project conceptualization to implementation.

#### Don’t Stop at Insights

Some HCD projects can run the risk of seeing the insights as an end, rather than a means to an end. When design projects are scoped with insights as an output, traditional global health actors, country governments, or other stakeholders must take the extra step to translate these insights into action to achieve outcomes. Programs that continued beyond insight generation to prototyping and actual implementation achieved greater success in the adoption of project recommendations.

Programs that continued beyond insight generation to prototyping and actual implementation achieved greater success in the adoption of project recommendations.

In A360 Ethiopia, the insights-gathering phase yielded findings similar to those found in the adolescent and youth sexual and reproductive health sector previously, such as “contraception is at odds with a girl’s identity and what is expected of her” or “proving fertility and having children is culturally revered.” The HCD process was more valuable in translating these insights and learnings into tangible programming opportunities. For example, from these insights, the team built rough prototypes of a baby cost calculator, a newlywed counseling session, a young wives club, junior health extension workers, and many more. Following insights generation with rapid solution prototyping helped to deepen learnings gleaned from the HCD process and make more actionable progress toward behavior change.

Conversely in Reimagining TA, the project ended with insights and concept generation, including 20 principles of good TA. The team originally planned for 4 workshops to move stakeholders through a design process. However, this proved unrealistic due to the complexity of the “problem” to be solved, the inconsistency of stakeholder attendance at the workshops, and the time frame allocated. Given the constraints, the team determined that the workshops could be better leveraged to develop a set of design principles to guide and govern TA planning and delivery. Implementation of the design principles in each country would have required additional funding and time but would have been more effective in achieving the intended impact of the program.

To address these challenges, first, the project itself should have been co-created with an emphasis on the iterative nature of the HCD process. The full project design, timeline, and funding allocation should have only been developed after defining the design challenge or questions. After understanding the complex nature of the design challenge, all actors in the process, including the funder, should have agreed to a flexible timeline and funding needed to co-create the vision, test solutions, implement, and evaluate.

#### Embed Design Skills Among Partners

The HCD process and related outputs are highly tailored to the needs of a target population that will shift over time. These projects illustrate how engaging partners throughout the project, and particularly during the insight-generation phase, builds capacity and buy-in to HCD methodology. Including additional budget and time to support the active development of these skills will better enable countries to respond to changing needs of the target population once the “project” has ended.

In V, Engage Design, the HCD consortium, partnered with PSI and Pangaea Zimbabwe AIDS Trust to scale V in Zimbabwe. This partnership focused specifically on building HCD capacity and the expertise needed to implement and adapt V independently after the Engage Design support ends. Engage Design sought to empower and inspire local ownership through capacity-building workshops and opportunities for program implementers to work alongside the design team at every stage of the project. PSI and Pangaea Zimbabwe AIDS Trust staff began as observers, then with coaching, gradually became cofacilitators and lead facilitators, working alongside Engage Design project staff. This collaborative approach and tapered support plan were also designed to improve the V product during implementation, demonstrating how HCD capacity building also benefits the quality of global health program outputs.

For Reimagining TA, given timeline and budget constraints, there was no time to build the HCD skills of in-country co-creation team members. Therefore, the solutions identified have not been implemented systematically.

#### Develop Design-Sensitive M&E Frameworks

Both design and traditional global health approaches are valuable when defining suitable M&E frameworks. These projects experienced success with both a more traditional global health approach for measuring longer-term outcomes and with a more design-led approach for iterative process measures. However, both cases required one group to adapt their traditional methods.

In A360 Ethiopia, taking a design-led approach to monitoring and evaluating the Smart Start program promoted greater adaptive management that was conducive to continually optimizing the program for scale-up across Ethiopia. Those familiar with traditional global health approaches might be tempted to try to rigorously monitor early prototyping phases and make decisions based on predefined indicators. However, in A360 Ethiopia, the team found it important to regard prototyping phases as formative research, while keeping the suite of indicators broad. For example, instead of only tracking how many girls were reached and how many girls adopted a contraception method in prototyping rounds, it was equally important to gather feedback on how girls were hearing about the program, how their partners felt about the program, and how long it was taking for providers to deliver a counseling session. Tracking this more expansive set of indicators led the team to make important refinements to optimize impact during scale-up (like cutting program components that were not adding value, shortening the lengthy curriculum, and creating new roles for community members recruiting girls). The ability to flexibly apply a mix of qualitative and quantitative methods to gather data early on was instrumental in helping the team to constantly learn and iterate on the program model.

For V, an independent impact evaluation failed to launch due to challenges in identifying a sufficiently rigorous methodology at a feasible cost. The evaluation of V in Zimbabwe was therefore designed to leverage existing, routine program monitoring systems. The learning objectives for V included first assessing acceptability and relevance with target end users and second, evaluating the feasibility of integrating V into existing oral PrEP service delivery. Impact will be assessed using PEPFAR’s standard performance indicators for oral PrEP. In addition, a learning agenda was developed, with questions designed to benefit both Zimbabwe and global communities (e.g., What are the similarities and differences between the experiences in South Africa and Zimbabwe? And what can we learn about applying this approach in other countries interested in V?). This blended approach of leveraging existing M&E systems alongside questions that can advance learnings related to HCD can support key decision makers in determining whether—and how—to scale programs like V.

## DISCUSSION

The 3 program experiences analyzed in this article offer lessons for planning, engaging, and applying individually. Additionally, several of these lessons are interrelated and illuminate insights for the project approach overall. Reflections from these projects suggest a virtuous cycle between the insights in each category. For example, the more that stakeholders, particularly country governments and end consumers, are engaged in an inclusive, participatory process, the greater their continued willingness and motivation to engage. Deeper engagement allows for more iteration and leads to higher quality, better-tailored outputs that are more likely to be sustainably used and scaled. Intentionally building HCD capacity among stakeholders over the course of the project strengthens this virtuous cycle by allowing the iterative process to continue once the “project” has ended. A360, V, and Reimagining TA in DRC were able to foster this cycle from the beginning and the solutions have continued to scale. Conversely, Reimagining TA in Nigeria was less able to undertake a participatory process from the start, which made securing country buy-in difficult and ultimately hindered implementation of project recommendations.

Intentionally building HCD capacity among stakeholders over the course of the project strengthens this virtuous cycle by allowing the iterative process to continue once the “project” has ended.

The ability to engender this virtuous cycle depends on another insight previously discussed: the importance of scoping projects appropriately to allow for co-creation, iteration, and HCD capacity building. Programs that seek to incorporate an HCD approach need to be scoped differently than traditional global health programs. The project conceptualization and scoping process itself needs to be more collaborative, and the budget and timeline need to be more flexible and sometimes be longer than traditional programs to allow for greater iteration. However, longer timelines are not always necessary; a nimbler HCD approach can be achieved by building in more built-in stage gates or pivot points for rapid prototyping and iteration at different stages of the process. A more inclusive scoping process may lead to a problem definition and output that is different from donor expectations and perceived as less directly focused on a funder’s mandate.

Yet, these projects exemplify that ultimately it helps to reduce rework and wasted resources and promote more sustained impact through investing in stakeholder buy-in and iteration upfront. In this way, HCD practices represent more inclusive and participatory approaches that should inspire us to take further steps toward decolonizing program development in global health; however, more can be done to further define where HCD is additive to those aspirations and where it is further exacerbating historical power imbalances (i.e., many HCD design agencies or teams are based in North America and Europe).

Of course, expanding the integration of HCD into more global health programs will require convincing funders and other decision makers that these programs are valuable. When considering a new approach, traditional global health stakeholders may perceive a higher risk of failure, especially when weighed against methods that are better understood and supported by well-documented evidence. Thus, there is a substantial burden of evidence required to persuade actors that this approach is not only beneficial but also that the risk of not having local ownership limits long-term benefits if it means projects will require ongoing grant funding.

The gold standard in global health programs is demonstrating cost-effectiveness; however, there is little publicly available information on the cost of these programs. Further, it is difficult to clearly break down costs for specific activities and phases, as budgets are allocated at a high level and multiple workstreams often take place in parallel. However, this type of estimation was attempted in a mid-term evaluation of A360, which found it cost between US$2 million—US$3.5 million to design the project approach.^31^ This included the cost to convene the consortium partners in the inception phase, as well as some costs from later phases for continued refinement. Further transparency and analysis of the true cost—and cost-effectiveness—of incorporating HCD into global health programs are needed to persuade others to adopt this approach.

Alternative approaches to measuring HCD’s contributions should also be considered. The results of the projects examined in this article demonstrate the value that both program implementers and program beneficiaries received. However, rigorous process evaluations to assess HCD’s contribution throughout programming implementation is an area that merits further investigation and investment, given their acceptance in global health implementation science programming. At the same time, the concept of value to the end user is increasingly being heralded to improve health services, and HCD brings a unique perspective on how best to define those value-based indicators. For example, end users may not only value clinical outcomes but also value improvements in quality and experience of care as well as the quality of life and well-being.^32^ Given the expense of rigorous, impact-level evaluations of clinical outcomes, defining HCD’s success in terms of how it can improve a patient’s perceptions of value presents a lower-cost alternative that aligns with HCD’s ethos of user-centric solutions. Further elaboration on best practices for M&E of HCD projects in global health is detailed in another article in this supplement.^33^

## CONCLUSION

The global community continues to seek solutions to achieve the ambitious SDGs for health. The experiences of V, A360 in Ethiopia, and Reimagining TA strongly suggest that integrating HCD into planning, engaging, and applying in global health programming could help traditional global health actors achieve better outcomes. HCD’s iterative and inclusive approach can produce more targeted and effective interventions while building local ownership and promoting more sustained impact. However, not every challenge is well suited for HCD, and programs need to be scoped and implemented thoughtfully to reap the benefits of an HCD approach and promote sustainability. A participatory, collaborative, and iterative co-creation approach from the project outset is important, as well as scoping the project in a way that supports an iterative approach throughout implementation. Further work is needed to better quantify the costs and benefits of integrating HCD methods to provide more evidence-based justification for the appropriate use of HCD. In the meantime, alternative approaches to characterizing the benefits of integrating an HCD approach should also be considered. As the global health field grapples with how to close the growing SDG gap, especially with the disruptions from COVID-19, HCD can be a powerful tool to generate progress toward these goals and more sustained impact.

## Notes

**Peer Reviewed**

## References

[1] Official Development Assistance. Accessed August 24, 2021. https://www.oecd.org/dac/financing-sustainable-development/development-finance-standards/official-development-assistance.htm

[2] WHO calls for reinvigorated action to fight malaria. World Health Organization. Accessed August 24, 2021. https://www.who.int/news/item/30-11-2020-who-calls-for-reinvigorated-action-to-fight-malaria

[3] Ensure healthy lives and promote well-being for all at all ages. United Nations Statistics Division. Accessed August 24, 2021. https://unstats.un.org/sdgs/report/2019/goal-03/

[4] Gates B Gates MF. COVID-19: a global perspective. Bill & Melinda Gates Foundation. September 2020. Accessed August 24, 2021. https://ww2.gatesfoundation.org/goalkeepers/report/2020-report/#GlobalPerspective

[5] Decade of action. United Nations. Accessed August 24, 2021. https://www.un.org/sustainabledevelopment/decade-of-action

[6] Glassman A Temin M. *Millions Saved: New Cases of Proven Success in Global Health*. Brookings Institution Press; 2016.

[7] Bernson J Zehrung D Gowda B Mulder M. Five years later: The Global Health Innovation Accelerator. PATH. December 12, 2019. Accessed August 24, 2021. https://www.path.org/articles/five-years-later-global-health-innovation-accelerator/

[8] Bao J Rodriguez DC Paina L Ozawa S Bennett S. Monitoring and evaluating the transition of large-scale programs in global health. Glob Health Sci Pract. 2015;3(4):591–605. 10.9745/GHSP-D-15-00221. 26681706 PMC4682584

[9] Yamey G. Scaling up global health interventions: a proposed framework for success. PLoS Med. 2011;8(6):e1001049. 10.1371/journal.pmed.1001049. 21738450 PMC3125181

[10] Shelton JD Waldman RJ. A journal for global health programming. Glob Health Sci Pract. 2013;1(1):3–4. 10.9745/GHSP-D-13-00001. 25276510 PMC4168564

[11] Design for Health. Accessed August 24, 2021. https://www.designforhealth.org

[12] HCDExchange. Accessed August 24, 2021. https://hcdexchange.org/

[13] IDEO.org. Accessed August 24, 2021. https://www.ideo.org/programs/billion-girls-colab

[14] Bazzano AN Martin J Hicks E Faughnan M Murphy L. Human-centred design in global health: a scoping review of applications and contexts. PLoS One. 2017;12(11):e0186744. 10.1371/journal.pone.0186744. 29091935 PMC5665524

[15] Tolley EE. *Traditional Socio-Behavioral Research and Human-Centered Design: Similarities, Unique Contributions and Synergies*. FHI360; 2017. Accessed August 24, 2021. https://www.theimpt.org/documents/reports/Report-HCD-BSS-Research.pdf

[16] Catalani C Green E Owiti P. A clinical decision support system for integrating tuberculosis and HIV care in Kenya: a human-centered design approach. PLoS One. 2014;9(8):e103205. 10.1371/journal.pone.0103205. 25170939 PMC4149343

[17] McCoy SI Fahey C Rao A Kapologwe N Njau PF Bautista-Arredondo S. Pilot study of a multi-pronged intervention using social norms and priming to improve adherence to antiretroviral therapy and retention in care among adults living with HIV in Tanzania. PLoS One. 2017;12(5):e0177394. 10.1371/journal.pone.0177394. 28486553 PMC5423659

[18] Kim S Piccinini D Mensah E Lynch M. Using a human-centered design approach to determine consumer preferences for long-lasting insecticidal nets in Ghana. Glob Health Sci Pract. 2019;7(2):160–170. 10.9745/GHSP-D-18-00284. 31249018 PMC6641816

[19] Kim S Myers CG Allen L. Health care providers can use design thinking to improve patient experiences. Harvard Business Review. August 31, 2017. Accessed February 24, 2021. https://hbr.org/2017/08/health-care-providers-can-use-design-thinking-to-improve-patient-experiences

[20] Roberts JP Fisher TR Trowbridge MJ Bent C. A design thinking framework for healthcare management and innovation. Healthc (Amst). 2016;4(1):11–14. 10.1016/j.hjdsi.2015.12.002. 27001093

[21] Brown T Wyatt J. Design thinking for social innovation Innovation. Stanford Social Innovation Review. 2010;8(1):30–35. Accessed August 24, 2021. https://ssir.org/articles/entry/design_thinking_for_social_innovation

[22] Why does design matter? Design for Health. Accessed August 24, 2021. https://www.designforhealth.org/understanding-design/why-does-design-matter

[23] Bass E Grant D. *AVAC Report 2019: Now What*? AVAC; 2019. Accessed August 24, 2021. https://www.avac.org/resource/avac-report-2019-now-what

[24] West GR Clapp SP Averill EMD Cates W Jr. Defining and assessing evidence for the effectiveness of technical assistance in furthering global health. Glob Public Health. 2012;7(9):915–930. 10.1080/17441692.2012.682075. 22606939 PMC3479625

[25] ActionAid. *Real Aid: Ending Aid Dependency*. ActionAid; 2011. Accessed August 24, 2021. https://reliefweb.int/sites/reliefweb.int/files/resources/Full_Report_2328.pdf

[26] Davis A Walker DG. On the path to UHC - global evidence must go local to be useful comment on "Disease Control Priorities Third Edition Is Published: A Theory of Change Is Needed for Translating Evidence to Health Policy." Int J Health Policy Manag. 2019;8(3):181–183. 10.15171/ijhpm.2018.118. 30980635 PMC6462195

[27] Bazzano AN Yan SD Martin J. Improving the reporting of health research involving design: a proposed guideline. BMJ Glob Health. 2020;5(2):e002248. 10.1136/bmjgh-2019-002248. 32133198 PMC7042569

[28] Andrawes L, Johnson T, Coleman M. Complexity in health: can design help support interdisciplinary solutions? Glob Health Sci Pract. 2021;9(Suppl 2). 10.9745/GHSP-D-21-00222 PMC8628505 34845045

[29] Irungu EM Baeten JM. PrEP rollout in Africa: status and opportunity. Nat Med. 2020;26(5):655–664. 10.1038/s41591-020-0872-x. 32405065

[30] Petersen ML Wang Y van der Laan MJ Guzman D Riley E Bangsberg DR. Pillbox organizers are associated with improved adherence to HIV antiretroviral therapy and viral suppression: a marginal structural model analysis. Clin Infect Dis. 2007;45(7):908–915. 10.1086/521250. 17806060 PMC2442040

[31] ITAD. A360 Evaluation: Mid-term Review Summary. ITAD; 2020. Accessed August 24, 2021. https://www.itad.com/wp-content/uploads/2020/02/A360-MTR-summary-2-1.pdf

[32] Leapfrog to Value. *Leapfrog to Value: How Nations Can Adopt Value-Based Care on the Path to Universal Health Coverage*. Global Development Incubator, Bill & Melinda Gates Foundation, USAID Center for Innovation and Impact, Rockefeller Foundation; 2019. Accessed August 24, 2021. https://www.usaid.gov/sites/default/files/documents/1864/Leapfrog_to_Value_Report.pdf

[33] Heller C LaFond A Murthy L. Methods and benefits of measuring human-centered design in global health. Glob Health Sci Pract. 2021;9(Suppl 2). 10.9745/GHSP-D-21-00207 PMC8628500 34845050

